# Boosting Turnover in the Triarylborane-Catalyzed Hydrogenation of *N*-Substituted Indoles via Olefin-to-Nitrogen Lewis Base Switching in H_2_-Cleavage Steps

**DOI:** 10.1021/prechem.4c00090

**Published:** 2024-12-18

**Authors:** Taiki Hashimoto, Masakazu Tanigawa, Kimitaka Kambe, Sensuke Ogoshi, Yoichi Hoshimoto

**Affiliations:** † Department of Applied Chemistry, Faculty of Engineering, Osaka University, Suita, Osaka 565-0871, Japan; ‡ Center for Future Innovation (CFi), Faculty of Engineering, Osaka University, Suita, Osaka 565-0871, Japan

**Keywords:** Indoles, Boranes, Hydrogenation, Frustrated Lewis pairs, H_2_ cleavage

## Abstract

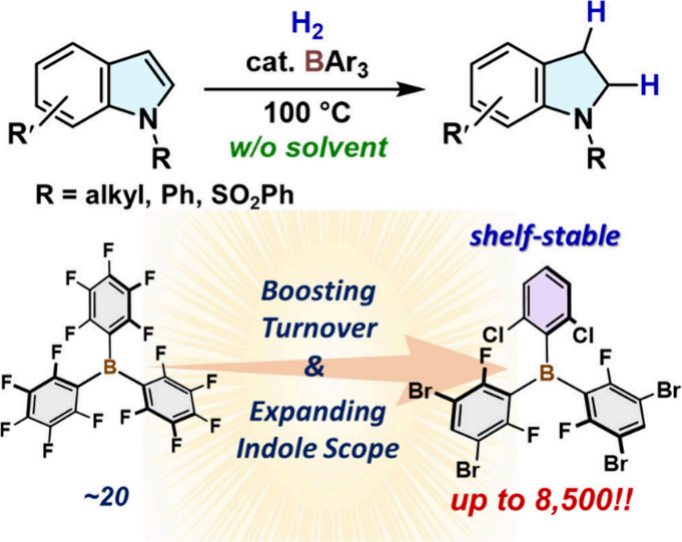

The shelf-stable heteroleptic borane B(2,6-Cl_2_C_6_H_3_)(3,5-Br_2_-2,6-F_2_C_6_H)_2_ (**B**^**7**^) efficiently catalyzes the solvent-free hydrogenation of various substituted indoles to indolines with an unprecedented turnover number of 8,500, which is more than 400-fold higher than that reported for B(C_6_F_5_)_3_ under diluted conditions. Mechanistic studies revealed that this hydrogenation proceeds via an olefin-to-nitrogen switching of Lewis bases involved in the H_2_-cleavage steps: initially, H_2_ cleavage is mediated by a frustrated Lewis pair (FLP) comprising the indole C3-carbon and boron atoms, which then switches to an FLP system comprising the indoline nitrogen and boron atoms after formation of the indoline. This study demonstrates the potential of relatively benign main-group elements for the catalytic synthesis of valuable N-containing molecules using H_2_.

## Introduction

**Figure 1:**
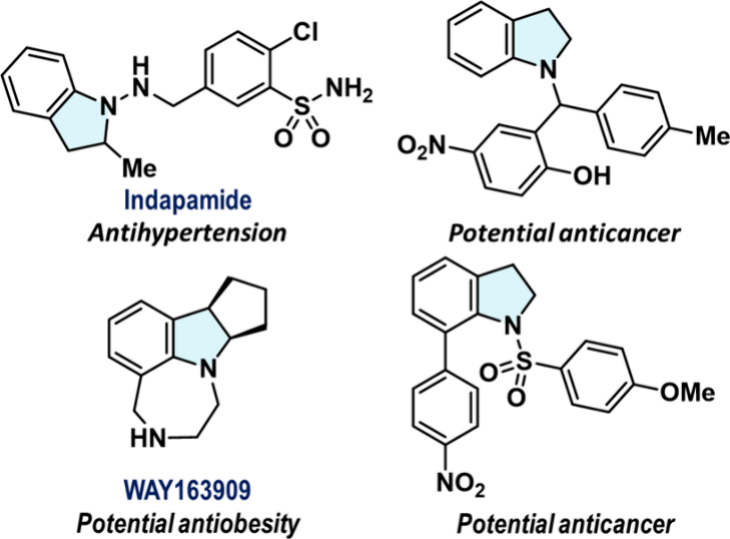
Selected examples of *N*-substituted indoline derivatives.

The construction of 2,3-dihydroindole moieties, also called indolines, has attracted much attention in the pharmaceutical field, owing to their unique structural features and biological properties. The indoline scaffold, which comprises a benzene ring fused with a pyrrolidine ring, offers key advantages in drug design; in particular, its noncoplanar structure enhances water solubility, and its benzene ring engages in hydrophobic interactions with protein residues. By virtue of these characteristics, indoline derivatives have found therapeutic applications, e.g., as anticancer, antitumor, and antihypertension agents (Figure 1).^1,2^ For the synthesis of indolines, the catalytic hydrogenation of indole derivatives stands out as an atom-economical approach that avoids the generation of stoichiometric waste associated with conventional reducing agents such as NaBH_3_CN.^3,4^ Various transition-metal catalysts have been developed for the hydrogenation of indoles, which achieve turnover numbers (TONs) in the hundreds.^1,5−8^ The use of triarylborane catalysts represents an attractive alternative to conventional methods using stoichiometric reductants or transition-metal catalysts because time- and cost-intensive removal of byproducts such as excess salts or potentially toxic metal residues is avoided. Therefore, considering the extensive progress in the triarylborane-catalyzed hydrogenation of unsaturated compounds,^9−11^ the triarylborane-catalyzed hydrogenation of indoles via heterolytic cleavage of H_2_ mediated by frustrated Lewis pairs (FLPs) can be considered as a promising strategy for the synthesis of indolines. However, the triarylborane-catalyzed hydrogenation of indole derivatives remains largely unexplored mainly because the indoline products may cause catalyst deactivation under conventional conditions.

**Figure 2:**
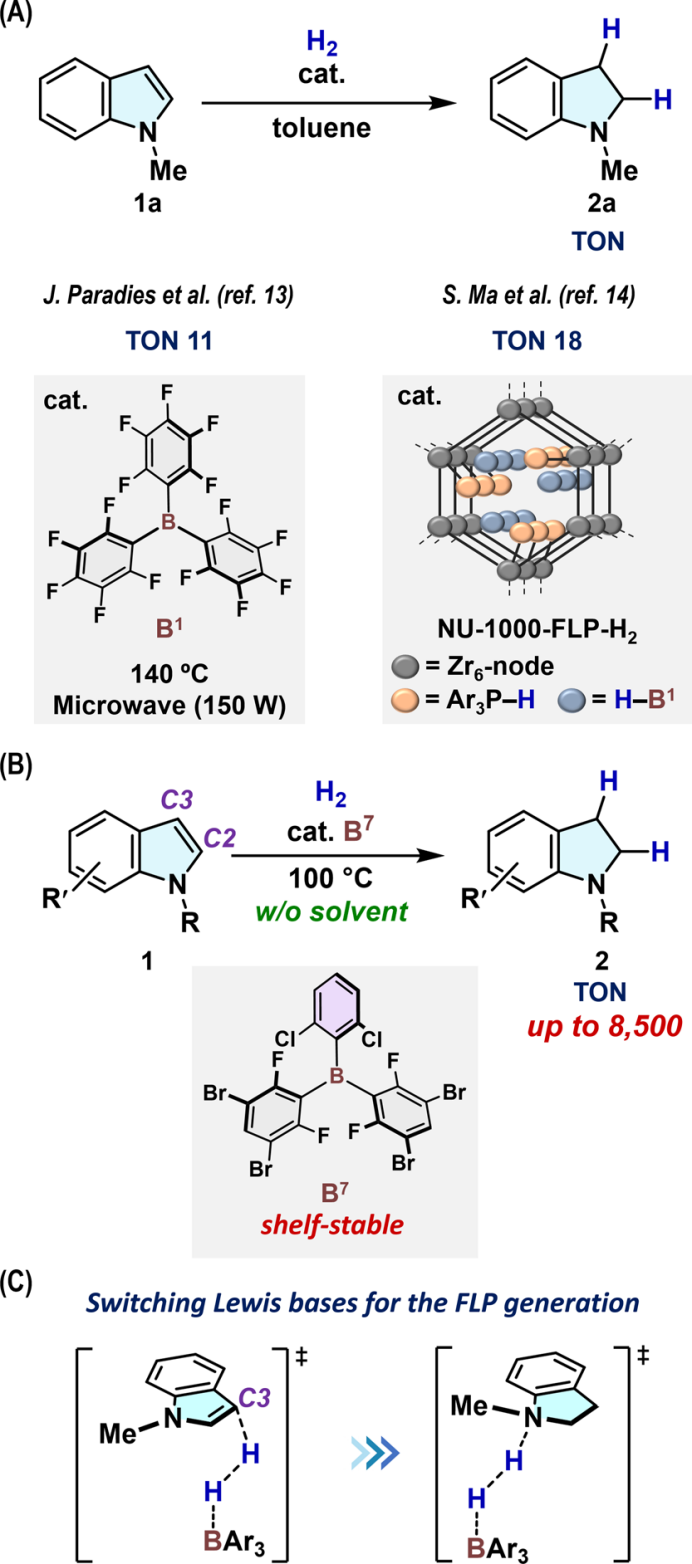
(A) Catalytic hydrogenation of 1-methylindole (**1a**) to give 1-methylindoline (**2a**); **B**^**1**^ catalyst under microwave condition (ref (13)); NU-1000-FLP-H_2_ catalyst (ref (14)). (B) **B**^**7**^-Catalyzed hydrogenation of *N*-substituted indoles under solvent-free conditions (*this work*). (C) Key olefin-to-nitrogen Lewis-base switching in FLP-mediated H_2_-cleavage steps.

In 2011, Stephan and co-workers reported the B(C_6_F_5_)_3_ (**B**^**1**^)-catalyzed hydrogenation of 1-methylindole (**1a**) and its derivatives in toluene using H_2_ (103 atm) at 80 °C;^12^ however, in 2016, Paradies and co-workers pointed out difficulties in reproducing the reported result. Instead, they demonstrated that the microwave-assisted hydrogenation of **1a** catalyzed by **B^1^** in toluene under H_2_ (4 atm) at 140 °C yielded a TON of 11 (left in Figure 2A).^13^ Recently, Niu, Lang, Ma, and co-workers reported the catalytic hydrogenation of *N*-methylindole derivatives in toluene using a Zr-based metal–organic framework (MOF; NU-1000-FLP-H_2_) containing an [(aryl)_3_P–H][H–**B**^**1**^] unit, achieving a TON of 18 (right in Figure 2A).^14^ However, the catalytic performance of such **B^1^**-based systems is substantially lower than that of transition-metal catalysts. Moreover, the air- and moisture-sensitivity of **B^1^** present considerable practical challenges. Given these limitations, we aimed to develop an efficient, robust, and practical triarylborane catalyst for the hydrogenation of indoles to valuable indolines (notably, the price of 1-methylindoline (**2a**) is 85 times higher than that of **1a**).^15^

Herein, we report the solvent-free hydrogenation of various substituted indoles catalyzed by shelf-stable B(2,6-Cl_2_C_6_H_3_)(3,5-Br_2_-2,6-F_2_C_6_H)_2_ (**B^7^**) under a H_2_ atmosphere (Figure 2B). Remarkably, **B^7^** achieves a TON of 8,500 in the hydrogenation of **1a**. The key to effectively enhancing the TON is the use of triarylboranes that resist irreversible proto-deboronation in the presence of both indolines and H_2_. Mechanistic studies revealed that the present hydrogenation involves an olefin-to-nitrogen switching of the Lewis bases during the formation of FLP species with boranes (Figure 2C).

## Results and Discussion

**Figure 3:**
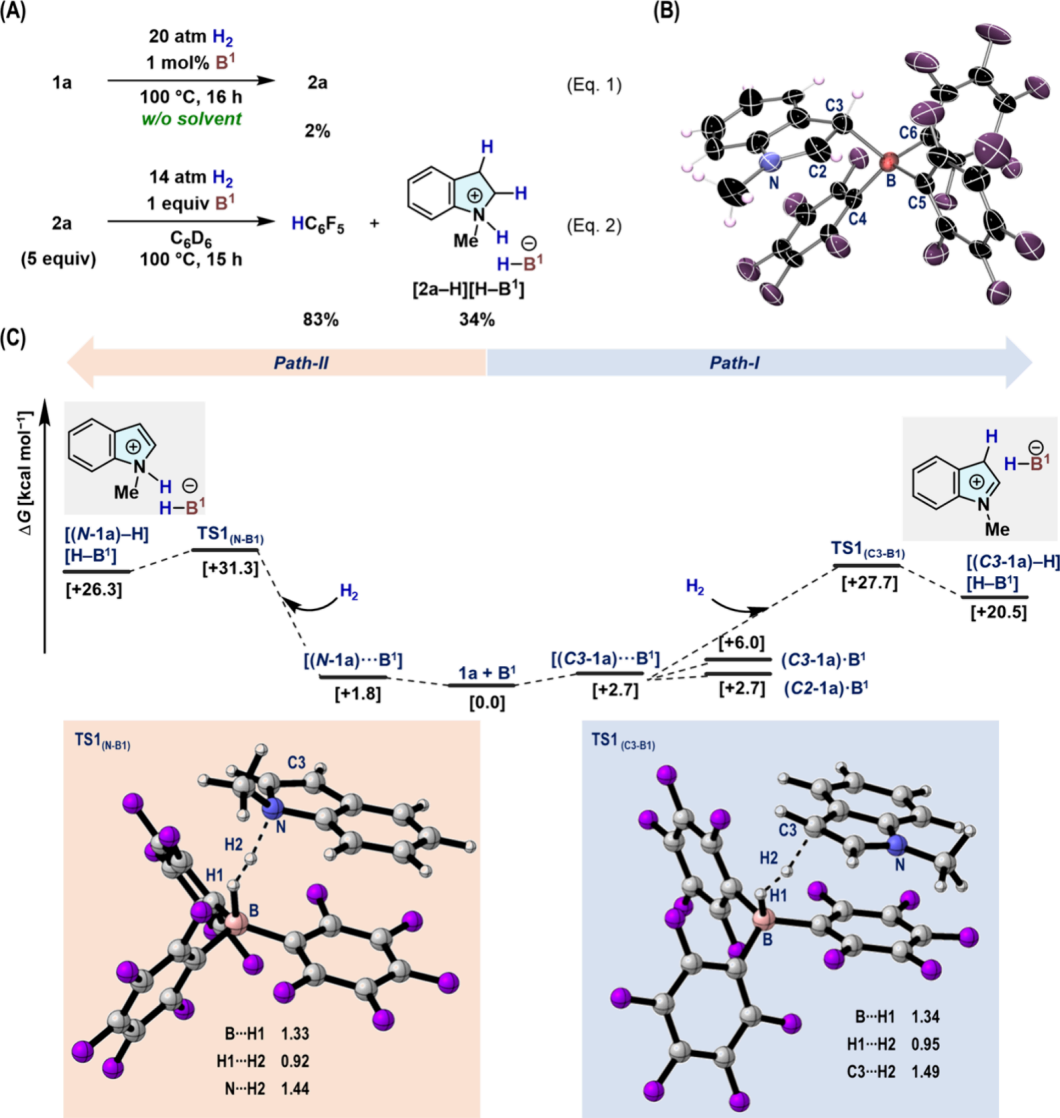
(A) **B**^**1**^-Catalyzed hydrogenation of **1a** using H_2_ (20 atm) at 100 °C under solvent-free conditions (eq 1); results for the reaction between **2a** (5 equiv) and **B**^**1**^ at 100 °C in C_6_D_6_ are also shown (eq 2). (B) Molecular structure of **(*****C3*****-1a)·B**^**1**^ (thermal ellipsoids: 30% probability), determined by single-crystal X-ray diffraction analysis. Selected bond lengths (Å) and angles (deg): B–C3 1.80 (1), N–C2 1.28 (1), C2–C3 1.46 (2), C3–B–C4 101.7 (8), C3–B–C5 107.6 (8), C3–B–C6 112.9 (8). (C) Theoretical analysis of the reaction among **1a**, **B**^**1**^, and H_2_, calculated at the M06-2X/Def2-TZVP//M06-2X/Def2-SVP level. Relative Gibbs energies (kcal mol^–1^) are given with respect to those of **[1a + B**^**1**^**]** (0.0 kcal mol^–1^). Structures of selected transition states **TS1**_**(C3–B1)**_ and **TS1**_**(N–B1)**_ are shown with selected distances (Å).

We began our investigation by examining the reasons behind the lack of examples of the **B^1^**-catalyzed hydrogenation of indoles, which contrasts with the successful application of **B^1^** in the hydrogenation of various N-containing unsaturated molecules such as imines, quinolines, and pyridines via FLPs containing N and B atoms.^9,10^ Indeed, **2a** was formed in only 2% yield (TON = 2) when conducting the hydrogenation of **1a** using 1 mol % **B**^**1**^ under solvent-free conditions at 100 °C (eq 1 in Figure 3A). Resconi and co-workers reported that the reaction between **1a** and **B**^**1**^ in CH_2_Cl_2_ at room temperature for more than 4 d led to the isolation of a **(*****C2*****-1a)·B**^**1**^ adduct.^16^ It was proposed that this adduct stems from the C3-to-C2 migration of the **B**^**1**^ unit from the initially formed **(*****C3*****-1a)·B**^**1**^, although experimental evidence that would support the generation of the latter C3-adduct was not presented. To investigate whether these adducts are related to the deactivation of **B**^**1**^, we attempted the isolation of **(*****C3*****-1a)·B**^**1**^. Eventually, a single crystal of **(*****C3*****-1a)·B**^**1**^ was obtained by the slow diffusion of *n*-hexane into a toluene solution of **1a** and **B**^**1**^ at −35 °C, enabling its structural characterization by single-crystal X-ray diffraction analysis, although the isolated yield could not be determined. As shown in Figure 3B, the formation of the C3–B bond was unambiguously confirmed. We subsequently optimized the gas-phase structure of **(*****C3*****-1a)·B**^**1**^ (+6.0 kcal mol^–1^) theoretically at the M06-2X/Def2-TZVP//M06-2X/Def2-SVP level. The relative Gibbs energies (Δ*G*) in Figure 3C are given with respect to [**1a** + **B**^**1**^] (0.0 kcal mol^–1^). Moreover, we optimized the structure of **(*****C2*****-1a)·B**^**1**^ (+2.7 kcal mol^–1^). These results indicate that the formation of **(*****C2*****-1a)·B**^**1**^ and **(*****C3*****-1a)·B**^**1**^ is endothermic, suggesting that these adducts are unlikely to affect the catalytic activity of **B**^**1**^. Importantly, we found that association complex **[(*****C3*****-1a)···B**^**1**^**]** (+2.7 kcal mol^–1^), which involves a pair of separated but preorganized boron 2p and olefinic π orbitals, was generated (Figure S7). Thus, **[(*****C3*****-1a)···B**^**1**^**]** can act as an olefin–borane FLP species for the mediation of the heterolytic cleavage of H_2_, although such species have so far remained elusive.^17,18^ In fact, a path leading to **[(*****C3*****-1a)–H][H–B**^**1**^**]** (+20.5 kcal mol^–1^) from H_2_ and **[(*****C3*****-1a)···B**^**1**^**]** via **TS1**_**(C3–B1)**_ (+27.7 kcal mol^–1^) was more favorable than a path for the cleavage of H_2_ with a nitrogen–borane FLP via **TS1**_**(N–B1)**_ (+31.3 kcal mol^–1^). Then, we turned our attention to exploring the potential role of **2a** in the decomposition of **B**^**1**^ (eq 2 in Figure 3A). Heating a mixture of **2a** and **B**^**1**^ in the presence of H_2_ (14 atm) at 100 °C in C_6_D_6_ resulted in the generation of HC_6_F_5_ in 83% yield via the irreversible proto-deboronation from a four-coordinated boron species including the **B**^**1**^ unit, with concomitant formation of **[2a–H][H–B**^**1**^**]**. Given that the generation of HC_6_F_5_ was negligible in the absence of H_2_ under identical conditions, the thermolysis of **[2a–H][H–B**^**1**^**]** is most likely responsible for the proto-deboronation yielding HC_6_F_5_. These results indicate that an effective borane catalyst must facilitate the proton/hydride transfer from **[2a–H][H–B**^**1**^**]** to another indole molecule before irreversible proto-deboronation occurs.

**Figure 4:**
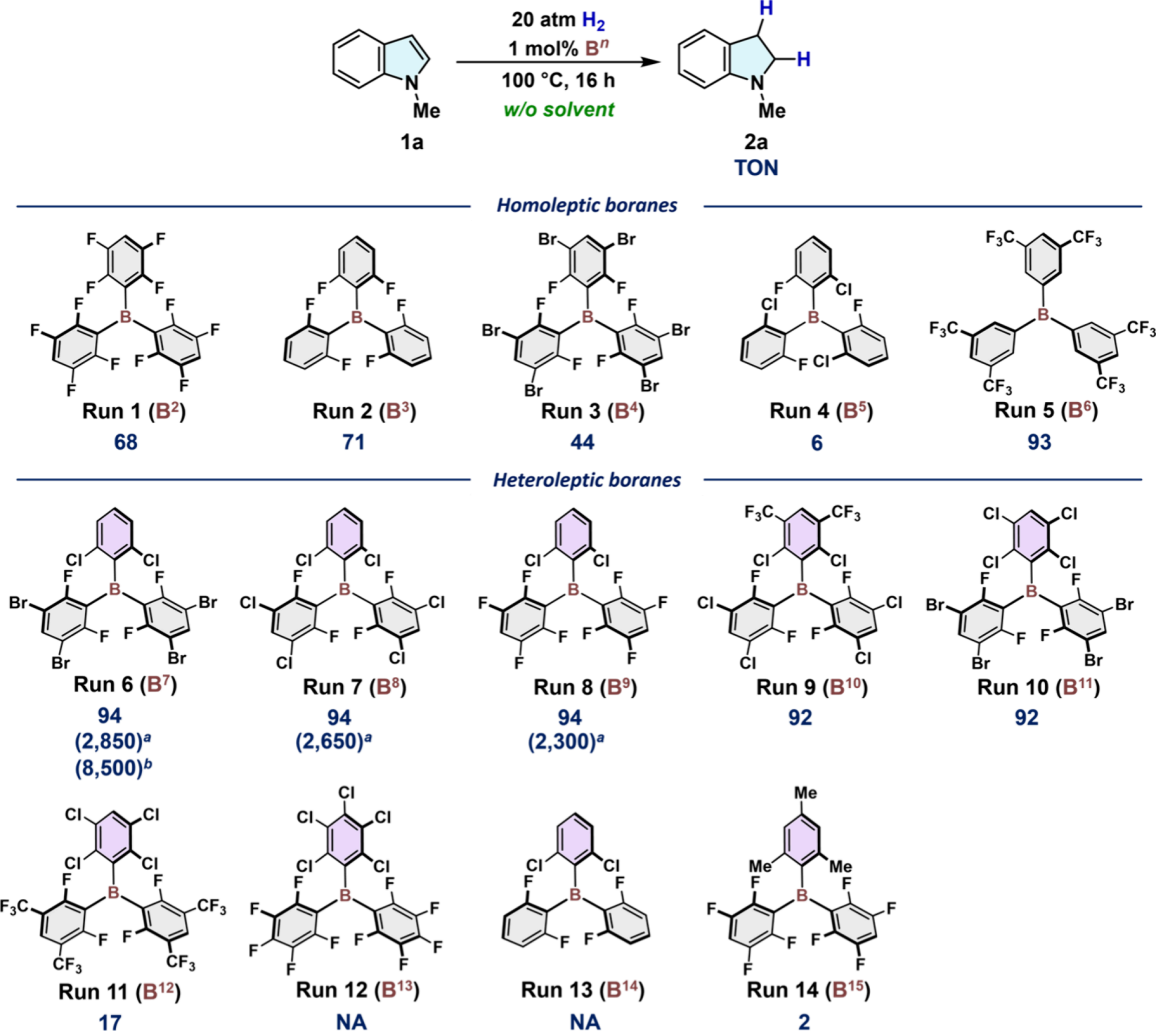
Catalyst screening under solvent-free conditions. General conditions: An autoclave (30 mL) was filled with **1a** (2.5 mmol), **B**^***n***^ (*n* = 2–15; 0.025 mmol), and tetradecane as an internal standard. After pressurization with H_2_ (20 atm), the mixture was stirred at 100 °C for 16 h. Conversion of **1a** and the catalyst turnover number (TON) were determined by GC analysis. [*a*] Catalyst turnover frequency (TOF in d^–1^) using 0.02 mol % **B**^***n***^ and H_2_ (60 atm). [*b*] TON after a period of 8 d using 0.01 mol % **B**^**7**^. H_2_ was pressurized to reach a total pressure of 60 atm once per day. NA: not available.

To optimize the triarylboranes for the solvent-free hydrogenation of **1a**, we carried out the reaction using 1 mol % boranes, including homoleptic (**B**^**2**^–**B**^**6**^)^19−23^ and heteroleptic (**B**^**7**^–**B**^**15**^)^24−29^ species (Figure 4). Decreasing the intrinsic Lewis acidity of the boron atom by substituting the C_6_F_5_ groups in **B**^**1**^ (TON = 2; Figure 3A) with 2,3,5,6-F_4_C_6_H groups (**B**^**2**^), 2,6-F_2_C_6_H_3_ groups (**B**^**3**^), and 3,5-Br_2_-2,6-F_2_C_6_H (**B**^**4**^) increased the TON to 68, 71, and 44, respectively (Figure 4, runs 1–3). Introduction of sterically demanding 2-Cl-6–F-C_6_H_3_ groups (B^5^) substantially decreased the TON to 6 (Figure 4, run 4). In contrast, B^6^, which bears sterically less hindered but strongly electron-withdrawing 3,5-(CF_3_)_2_C_6_H_3_ groups showed excellent catalytic activity (TON = 93; Figure 4, run 5), although its high sensitivity to air and moisture limits the scope of practical applications.

Recently, we have demonstrated that heteroleptic triarylboranes that bear 2,6-Cl_2_-aryl groups efficiently catalyze the hydrogenation of quinolines and *in situ*-generated imines, even in the presence of Lewis bases such as CO_2_ and H_2_O.^24,26^ The shelf-stable boranes **B^7^** and **B^8^** exhibited high activity (TON = 94; Figure 4, runs 6 and 7), comparable to that of the more moisture-sensitive **B^9^** (Figure 4, run 8). Upon slightly modifying the 2,6-Cl_2_C_6_H_3_ group in **B**^**7**^ and **B**^**8**^ by introducing *meta*-CF_3_ (**B**^**10**^) or *meta*-Cl (**B**^**11**^) groups, high catalytic activity was maintained to exhibit TONs of 92 (Figure 4, runs 9 and 10), while these boranes are synthetically more challenging than **B**^**7**^ and **B**^**8**^. However, replacing the *meta*-Br atoms in **B^11^** with *meta*-CF_3_ groups remarkably decreased the catalytic activity, whereby **B^12^** furnished **2a** in only a 17% yield (Figure 4, run 11). Boranes **B**^**13**^ and **B**^**14**^ did not catalyze the hydrogenation of **1a** (Figure 4, runs 12 and 13), even though the local environment surrounding the B atoms is nearly identical to that of **B**^**7**^–**B**^**11**^. Replacing the 2,6-Cl_2_-aryl groups with a 2,4,6-Me_3_C_6_H_2_ substituent (**B**^**15**^) was also unsuccessful (TON = 2; Figure 4, run 14).

Given their synthetic accessibility and practical utility, we compared the catalytic activity of **B**^**7**^–**B**^**9**^ by measuring their turnover frequencies (TOFs) using 0.02 mol % catalyst loading under H_2_ (60 atm). The TOF values increased from 2,300 d^–1^ for **B**^**9**^ (*meta*-F) to 2,650 d^–1^ for **B**^**8**^ (*meta*-Cl), and 2,850 d^–1^ for **B**^**7**^ (*meta*-Br), demonstrating that remote back strain given by the *meta*-substituents exerts a considerable influence on the catalyst performance and robustness.^30^ Notably, when the solvent-free hydrogenation of **1a** was conducted with 0.01 mol % **B^7^** over 8 d with daily recharging of H_2_ (60 atm), the TON reached 8,500, demonstrating the practical potential of main-group catalysis for such hydrogenation reactions.

**Figure 5:**
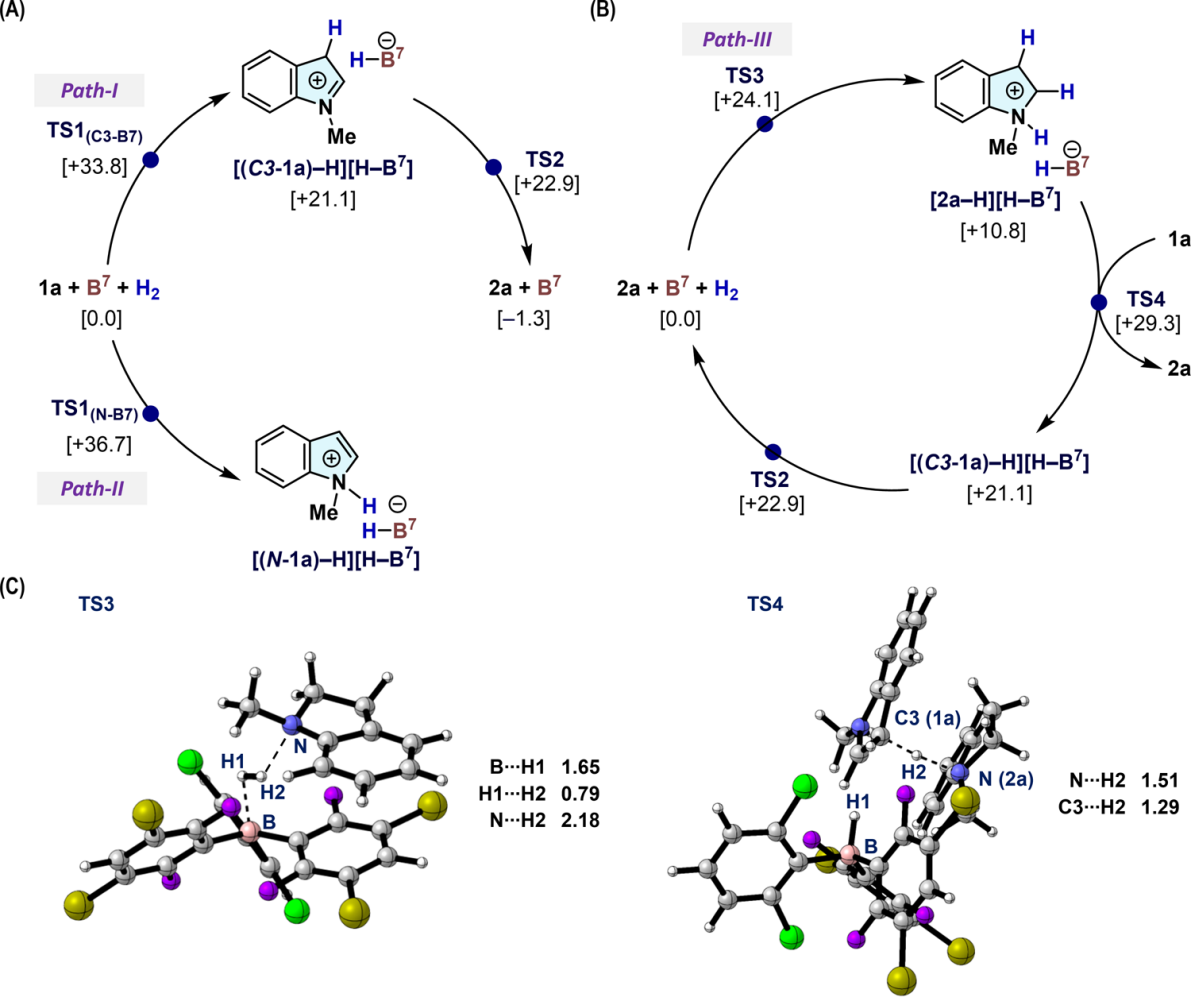
Plausible reaction mechanisms for the hydrogenation of **1a** with **B**^**7**^ via (A) **TS1**_**(C3–B7)**_/**TS1**_**(N–B7)**_ and (B) **TS3**. Relative Gibbs energies (kcal mol^–1^), calculated at the M06-2X/Def2-TZVP//M06-2X/Def2-SVP//gas-phase level, are given with respect to **[1a + 2a + B**^**7**^**+ H**_**2**_**]**, while the initial state is shown as (A) **[1a + B**^**7**^**+ H**_**2**_**]** or (B) [**2a** + **B**^**7**^**+ H**_**2**_] for clarity. (C) Optimized molecular structures of **TS3** and **TS4** with selected geometrical parameters (in Å).

Figure 5 illustrates plausible reaction mechanisms for the **B^7^**-catalyzed hydrogenation of **1a** to **2a**, where the Δ*G* values are referenced to **[B**^**7**^**+ 1a + 2a + H**_**2**_**]** (0.0 kcal mol^–1^). According to our findings with **B**^**1**^ (Figure 3C), we considered two possible paths for the heterolytic cleavage of H_2_ mediated by **B**^**7**^-based FLP species, i.e., one involving the olefinic C3 atom (path-I) and another involving the indole nitrogen atom (path-II) (Figure 5A). We confirmed that path-I, which affords **[(*****C3*****-1a)–H][H–B**^**7**^**]** (+21.1 kcal mol^–1^) via **TS1**_**(C3–B7)**_ (+33.8 kcal mol^–1^), would be favored over path-II, which furnishes **[(*****N*****-1a)–H][H–B**^**7**^**]** (+30.3 kcal mol^–1^) via **TS1**_**(N–B7)**_ (+36.7 kcal mol^–1^). Subsequently, in path-I, hydride migration smoothly takes place via **TS2** (+22.9 kcal mol^–1^) to yield **2a**. Once **2a** is generated, path-III, which involves H_2_ activation with indoline nitrogen and **B**^**7**^, becomes plausible, as evident from the decomposition of **B**^**1**^ from **[2a–H][H–B**^**1**^**]** (eq 2 in Figure 3B). DFT calculations suggested that path-III takes place predominantly to afford **[2a–H][H–B**^**7**^**]** (+10.8 kcal mol^–1^) via **TS3** (+24.1 kcal mol^–1^), followed by an intermolecular proton transfer via **TS4** (+29.3 kcal mol^–1^) to another molecule of **1a** to generate **[(*****C3*****-1a)–H][H–B**^**7**^**]** with concomitant regeneration of **2a**. The total energy barrier for overcoming these steps (Δ*G*^‡^ = +29.3 kcal mol^–1^) is lower than that of path-I (Δ*G*^‡^ = +33.8 kcal mol^–1^), which indicates that the catalytic cycle shifts from path-I to path-III with increasing concentration of **2a**. The increase in the concentration of **2a** under the present conditions (H_2_: 60 atm) may also induce the proto-deboronation of triarylboranes by promoting the generation of **[2a–H][H–B**^***n***^**]**. Nevertheless, **[2a–H][H–B**^**7**^**]** would be effectively destabilized through back strain between the 2,6-Cl_2_C_6_H_3_ and 3,5-Br_2_-2,6-F_22_C_6_H groups, thus preventing the proto-deboronation and facilitating the subsequent hydride transfer.

**Figure 6:**
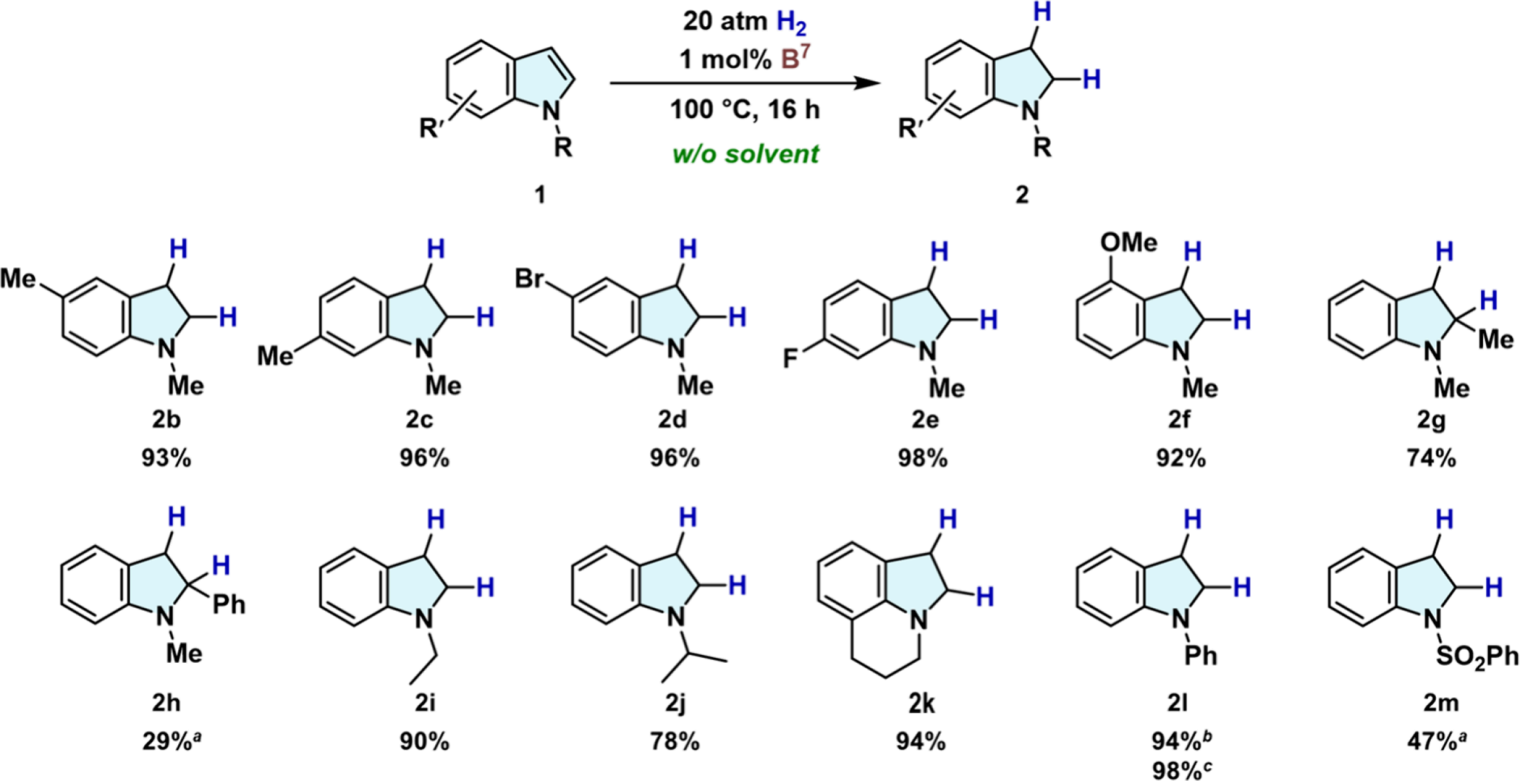
Triarylborane-catalyzed synthesis of substituted indolines **2** via hydrogenation of indoles **1** under solvent-free conditions. General conditions: An autoclave (30 mL) was filled with **1a** (2.5 mmol), and **B**^**7**^ (0.025 mmol). Tetradecane or dodecane were used as an internal standard. After pressurization with H_2_ (20 atm), the mixture was stirred at 100 °C for 16 h. The product yield was determined via GC analysis. ^*a*^1 mol % **B**^**1**^ was used. ^*b*^A mixture of **B**^**1**^ and **B**^**7**^ (1 mol % each) was used. ^*c*^ A mixture of **B**^**7**^ and **2a** (2 mol % each) was used.

Finally, we explored the applicability of shelf-stable **B**^**7**^ to the solvent-free hydrogenation of substituted indoles **1** to yield indolines **2** (Figure 6). Various 1-methylindole derivatives (**1b**–**1f**) were converted to the corresponding indolines (**2b**–**2f**) in excellent yield, albeit that **2g** was obtained from C2-methylated **1g** (mp ∼55 °C) in merely 74% yield owing to the increased steric hindrance at the C2 position. Although **B**^**7**^ did not work well, the use of **B**^**1**^ gave 1-methyl-2-phenylindoline (**2h**) in a 29% yield. It should be emphasized here that the present **B**^**7**^-catalyzed system can also be applied to *N*-substituted indoles (**1i**–**1m**), which have remained unexplored as substrates for main-group-based catalytic systems. In the presence of 1 mol % **B**^**7**^, indolines bearing *N*-Et (**2i**) and *N*-^*i*^Pr (**2j**) groups were synthesized in 90% and 78% yield, respectively. Tricyclic lilolidine (mp ∼85 °C) **1k** was also suitable for the present solvent-free system and afforded **2k** in 94% yield. Conversely, **B^7^** showed low catalytic activity for the hydrogenation of 1-phenyl-1*H*-indole (**1l**), giving **2l** in only 15% yield, even when using 2 mol % catalyst. This low reactivity can most likely be attributed to the decreased Lewis basicity at the C3 position, which would impede the H_2_ activation with **B**^**7**^ in the initial catalytic cycle (*cf*. path-I in Figure 5). In this context, a mixed catalyst system of **B^1^** and **B^7^** (1 mol % each) substantially increased the yield of **2l** to 94%, improving the result with 2 mol % **B^1^** alone (87%), which can be ascribed to **B**^**1**^ predominantly mediating the H_2_ cleavage with the indole C3 atom and the **2a**/**B**^**7**^ pair promoting the following process. Moreover, **2l** was obtained in 98% yield when a mixture of **B**^**7**^ and **2a** (2 mol % each) was applied, demonstrating the critical effect of shifting the H_2_-cleavage mechanism. Indole **1m** (mp ∼78 °C), which contains a strongly electron-withdrawing SO_2_Ph moiety, was also suitable and afforded **2m** in 47% yield in the presence of 1 mol % **B**^**1**^. Therefore, **B^7^** is more suitable for the hydrogenation of indoles that bear *N*-electron-donating alkyl groups and/or sterically less hindered nitrogen centers, whereas **B^1^** is preferable for substrates with *N*-electron-withdrawing or bulky substituents, as these groups reduce the risk of proto-deboronation.

## Conclusions

In summary, we have demonstrated that shelf-stable B(2,6-Cl_2_C_6_H_3_)(3,5-Br_2_-2,6-F_2_C_6_H)_2_ (**B**^**7**^) efficiently catalyzes the solvent-free hydrogenation of various substituted indoles to indolines. Remarkably, we achieved an unprecedented turnover number (TON) of 8,500 in the main-group-catalyzed hydrogenation of 1-methylindole (**1a**), representing a more than 400-fold improvement over hitherto reported systems based on B(C_6_F_5_)_3_. Mechanistic studies revealed that the hydrogenation of **1a** involves an olefin-to-nitrogen switching of Lewis bases in the critical H_2_-cleavage steps. Specifically, the initial H_2_ activation is mediated by a frustrated Lewis pair (FLP) comprising the indole C3-carbon and boron atoms, while an FLP species comprising the indoline nitrogen and boron atoms promotes the subsequent H_2_ activation leading to indoline **2a**. Thus, the design of an effective triarylborane should prevent borane decomposition, which occurs via thermally induced proto-deboronation from [indoline–H][H–borane] species. In this context, judiciously designed **B**^**7**^ showed notable applicability for the synthesis of *N*-substituted indolines, which have rarely been synthesized by using main-group-based catalysts. Therefore, this work demonstrates the high potential of triarylboranes as catalysts for the hydrogenation of unsaturated molecules to produce valuable nitrogen-containing compounds.

## Supporting Information Available

Full details pertaining to experimental methods, identification of compounds, and DFT calculations (PDF)
Computed Cartesian coordinates for optimized structures (XLSX)
Crystallographic data (CIF)

## Supplementary Material

pc4c00090_si_002.xlsx

pc4c00090_si_003.cif
