# Supplementary material for: Boosting Turnover in the Triarylborane-Catalyzed Hydrogenation of N-Substituted Indoles via Olefin-to-Nitrogen Lewis Base Switching in H2-Cleavage Steps
Source: Precis Chem. 2024 Dec 18;3(3):128–34. doi: 10.1021/prechem.4c00090 (PMC11938162; doi:10.1021/prechem.4c00090)
Supplement: Supplementary file 1 — pc4c00090_si_001.pdf [file pc4c00090_si_001.pdf]

Supporting Information

Boosting Turnover in the Triarylborane-Catalyzed Hydrogenation of *N*-Substituted Indoles via Olefin-to-Nitrogen Lewis-Base Switching in H_2-Cleavage Steps

Taiki Hashimoto,[a] Masakazu Tanigawa,[a] Kimitaka Kambe,[a] Sensuke Ogoshi,[a] and Yoichi Hoshimoto*[a,b]

[a] Department of Applied Chemistry, Faculty of Engineering, Osaka University, Suita, Osaka 565-0871, Japan

[b] Center for Future Innovation (CFi), Faculty of Engineering, Osaka University, Suita, Osaka 565-0871, Japan.

E-mail to: hoshimoto@chem.eng.osaka-u.ac.jp

Table of Contents

[1] General considerations

[2] Materials

[3] Crystallization of (*C3*-1a)·B[1]

[4] Screening of catalysts in hydrogenation of 1a

4-1. Reactions with 1 mol% triarylborane catalysts

4-2. Catalyst turnover frequencies exhibited by triarylboranes

4-3. Catalyst turnover number exhibited by B[7]

[5] Stoichiometric reaction between B[1] and 2a in the presence of H_2

[6] Scope of indoles

[7] Theoretical studies

7-1. Computational details

7-2. Selected frontier molecular orbitals (FMOs) for [(*C3*-1a)···B[1]]

7-3. Plausible pathways for the reaction among 1a, B[1] and H_2

7-4. Plausible reaction mechanisms for the hydrogenation of 1a with B[7]

[8] References for the Supporting Information

[1] General considerations

Unless otherwise noted, all manipulations were conducted under a nitrogen atmosphere using standard Schlenk line or glovebox techniques. ^{1}H, ^{11}B, and ^{19}F NMR spectra were recorded on a Bruker AVANCE III 400. The chemical shifts (ppm) in the ^{1}H NMR spectra were recorded relative to tetramethylsilane or residual protonated solvent (C_6D_5H (δ 7.16), $CHCl_3$ (δ 7.26)). The chemical shift in the ^{11}B NMR spectra was recorded relative to $BF_3{\cdot}(OCH_2CH_3)$ as an external standard. The chemical shifts in the ^{19}F NMR spectra were recorded relative to α,α,α-trifluorotoluene (δ −65.64). Single crystal X-ray diffraction data were collected with a Rigaku XtaLAB Synergy diffractometer equipped with a HyPix-6000HE detector. Analytical gas chromatography (GC) was carried out on a Shimadzu GC-2025 gas chromatograph, equipped with a flame ionization detector.

[2] Materials

All commercially available reagents including super-dehydrated solvents (*n*-hexane, toluene) were purchased from Sigma Aldrich, Angene Chemical, Tokyo Chemical Industry (TCI) and FUJIFILM Wako Pure Chemical Corporation, and used as received. Benzene-d_6 was distilled from sodium benzophenone ketyl prior to use. $CDCl_3$ was stored over molecular sieves (4 Å). 1-methylindole (**1a**), tetradecane, and dodecane were purchased from TCI or Angene Chemical, and used after distillation over CaH_2. Some of the indole derivatives (**1b**, **1c**, **1e**, **1f**, **1i**, and **1j**) were prepared by following the reported procedures.[S1] H_2 gas was purchased from Sumitomo Seika Chemicals Company, and used as received. Note that this gas includes some impurities, as shown in Table S1.

	Impurity				
	N_2	O_2	CO	CO_2	H_2
H_2	<200	<50	<1	<1	-

Table S1. Impurities contaminated in H_2 (shown in ppm).

Metrical data for the solid-state structures is available from Cambridge Crystallographic Data Centre: CCDC2401790 ((***C3*-1a)·B^1**).

[3] Crystallization of (*C3*-1a)·B[1]

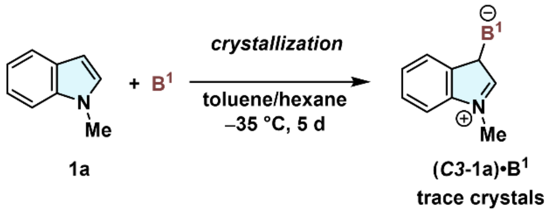

Figure S1. Crystallization of **(*C3*-1a)·B[1]**.

To a solution of **B[1]** (155 mg, 0.303 mmol, 0.3 M in toluene) was added **1a** (38.7 mg, 0.295 mmol). The reaction mixture was stirred for 5 min, and hexane was added over the resultant solution (ca. 2 mL). A single-crystal suitable for X-ray diffraction analysis was obtained by crystallization from toluene/hexane at −35 °C after the period of 5 d. X-ray data for **(*C3*-1a)·B1** (Figure S2): M = 643.16, colorless, monoclinic, $P2_1$ (#4), a = 7.5418(10) Å, b = 16.6471(13) Å, c = 9.6601(10) Å, α = 90°, β = 106.581(12)°, γ = 90°, V = 1162.4(2) Å^3, Z = 2, *Dcalcd* = 1.838 g/cm^3, T = −150 °C, R_1 (wR_2) = 0.1004 (0.2621).

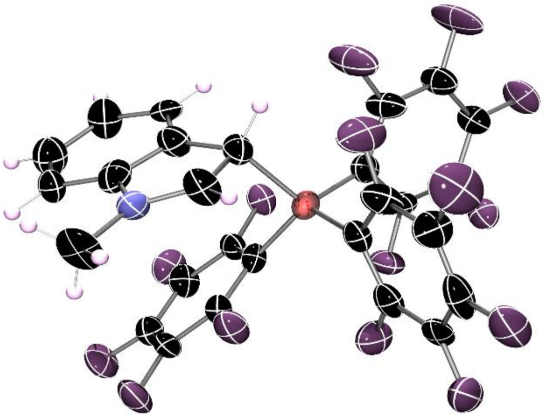

Figure S2. Molecular structure of **(*C3*-1a)·B[1]** with ellipsoids set at 30% probability

[4] Screening of catalysts in hydrogenation of 1a

4-1. Reactions with 1 mol% triarylborane catalysts (Eq.1 in Figure 3a and runs 1–14 in Figure 4)

General: A 10 mL autoclave was charged with **1a** (ca. 2.5 mmol) and borane (ca. 0.025 mmol; 1 mol%). Tetradecane was added as an internal standard. Once sealed, the autoclave was pressurized with H_2 (20 atm) and heated at 100 °C for 16 h. After degassing at room temperature, the yield of **2a** was determined by the GC analysis.

Eq. 1 in Figure 3a: Followed by the general procedure, **1a** (324 mg; 2.47 mmol), **B[1]** (13.0 mg; 0.0254 mmol), and tetradecane (229 mg; 1.15 mmol) were employed, giving **2a** in 2% GC yield.

Run 1 in Figure 4: Followed by the general procedure, **1a** (331 mg; 2.52 mmol), **B**^{2} (11.4 mg; 0.0249 mmol), and tetradecane (233 mg; 1.17 mmol) were employed, giving **2a** in 68% GC yield.

Run 2 in Figure 4: Followed by the general procedure, **1a** (326 mg; 2.49 mmol), **B**^{3} (8.3 mg; 0.024 mmol), and tetradecane (230 mg; 1.16 mmol) were employed, giving **2a** in 71% GC yield.

Run 3 in Figure 4: Followed by the general procedure, **1a** (331 mg; 2.52 mmol), **B**^{4} (21.5 mg; 0.0261 mmol), and tetradecane (232 mg; 1.17 mmol) were employed, giving **2a** in 44% GC yield.

Run 4 in Figure 4: Followed by the general procedure, **1a** (326 mg; 2.49 mmol), **B**^{5} (9.9 mg; 0.025 mmol), and tetradecane (236 mg; 1.19 mmol) were employed, giving **2a** in 6% GC yield.

Run 5 in Figure 4: Followed by the general procedure, **1a** (337 mg; 2.57 mmol), **B**^{6} (16.6 mg; 0.0255 mmol), and tetradecane (227 mg; 1.14 mmol) were employed, giving **2a** in 93% GC yield.

Run 6 in Figure 4: Followed by the general procedure, **1a** (335 mg; 2.55 mmol), **B**^{7} (17.7 mg; 0.0253 mmol), and tetradecane (229 mg; 1.15 mmol) were employed, giving **2a** in 94% GC yield.

Run 7 in Figure 4: Followed by the general procedure, **1a** (336 mg; 2.56 mmol), **B**^{8} (13.3 mg; 0.0255 mmol), and tetradecane (231 mg; 1.16 mmol) were employed, giving **2a** in 94% GC yield.

Run 8 in Figure 4: Followed by the general procedure, **1a** (339 mg; 2.58 mmol), **B**^{9} (12.1 mg; 0.0266 mmol), and tetradecane (234 mg; 1.18 mmol) were employed, giving **2a** in 94% GC yield.

Run 9 in Figure 4: Followed by the general procedure, **1a** (334 mg; 2.55 mmol), **B**^{10} (17.3 mg; 0.0277 mmol), and tetradecane (236 mg; 1.19 mmol) were employed, giving **2a** in 92% GC yield.

Run 10 in Figure 4: Followed by the general procedure, **1a** (335 mg; 2.55 mmol), **B**^{11} (19.6 mg; 0.0255 mmol), and tetradecane (233 mg; 1.17 mmol) were employed, giving **2a** in 92% GC yield.

Run 11 in Figure 4: Followed by the general procedure, **1a** (329 mg; 2.51 mmol), **B**^{12} (18.2 mg; 0.0251 mmol), and tetradecane (234 mg; 1.18 mmol) were employed, giving **2a** in 17% GC yield.

Run 12 in Figure 4: Followed by the general procedure, **1a** (328 mg; 2.50 mmol), **B**^{13} (15.1 mg; 0.0254 mmol), and tetradecane (232 mg; 1.17 mmol) were employed, not giving **2a**.

Run 13 in Figure 4: Followed by the general procedure, **1a** (327 mg; 2.49 mmol), $\mathbf{B^{14}}$ (9.6 mg; 0.025 mmol), and tetradecane (232 mg; 1.17 mmol) were employed, not giving **2a**.

Run 14 in Figure 4: Followed by the general procedure, **1a** (324 mg; 2.47 mmol), $\mathbf{B^{15}}$ (10.4 mg; 0.0243 mmol), and tetradecane (228 mg; 1.15 mmol) were employed, giving **2a** in 2% GC yield.

4-2. Catalyst turnover frequencies exhibited by triarylboranes ($\mathbf{B^7}$–$\mathbf{B^9}$; runs 1–3 in Figure S3)

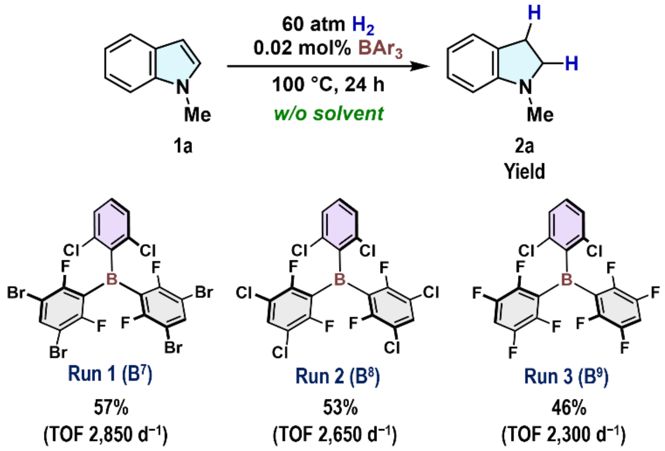

Figure S3. Catalytic solvent-free hydrogenation of **1a** using $\mathbf{B^7}$–$\mathbf{B^9}$.

General: A 30 mL autoclave was charged with **1a** (ca. 100 mmol), and borane (ca. 0.020 mmol; 0.02 mol%). Tetradecane was added as an internal standard. Once sealed, the autoclave was pressurized with H_2 (60 atm) and heated at 100 °C for 24 h. After degassing at room temperature, the yield of **2a** was determined by GC analysis.

Run 1: Followed by the general procedure, **1a** (13.0 g; 99.1 mmol), $\mathbf{B^7}$ (14.0 mg; 0.0200 mmol), and tetradecane (945 mg; 4.76 mmol) were employed, giving **2a** in 57% GC yield.

Run 2: Followed by the general procedure, **1a** (13.1 g; 99.9 mmol), $\mathbf{B^8}$ (10.6 mg; 0.0204 mmol), and tetradecane (1.00 g; 5.04 mmol) were employed, giving **2a** in 53% GC yield.

Run 3: Followed by the general procedure, **1a** (13.0 g; 99.1 mmol), $\mathbf{B^9}$ (9.0 mg; 0.020 mmol), and tetradecane (948 mg; 4.78 mmol) were employed, giving **2a** in 46% GC yield.

4-3. Catalyst turnover number exhibited by B^7 (Figure S4)

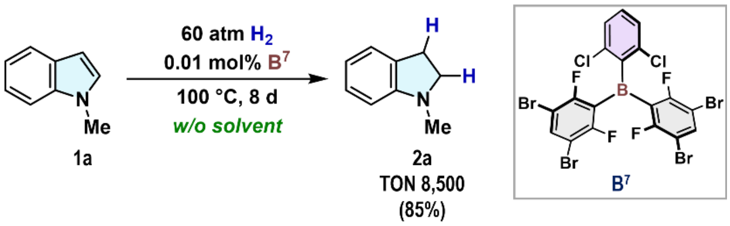

Figure S4. **B**[7]-catalyzed solvent-free hydrogenation of **1a**.

A 30 mL autoclave was charged with **1a** (26.1 g, 199 mmol) and **B**[7] (14.1 mg, 0.0202 mmol; 0.01 mol%). Tetradecane (2.03 g; 10.2 mmol) was added as an internal standard. Once sealed, the autoclave was pressurized with H_2 (60 atm) and heated at 100 °C. Through this experiment, we repeatedly pressurized H_2 to reach the total pressure of 60 atm at room temperature once a day. After heating for 8 d, **2a** was afforded in 85% GC yield (Figure S4).

[5] Stoichiometric reaction between B[1] and 2a in the presence of H_2

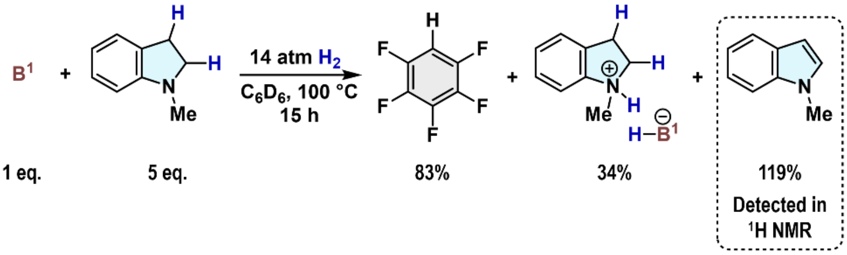

Figure S5. Stoichiometric reaction between **B**[1] and **2a** in the presence of H_2.

A high-pressure valved NMR tube (TCI S-5-500-HW-EX1-HPV-7; *V* = 1.8 mL) was charged with **2a** (33.8 mg, 0.254 mmol), **B**[1] (25.7 mg, 0.0502 mmol), and 1,3-dibromo-4,6-difluorobenzene (13.3 mg, 0.0489 mmol; an internal standard). Once sealed, the NMR tube was pressurized with H_2 (14 atm) and heated at 100 °C for 15 h. Then the ^{1}H, ^{11}B, and ^{19}F NMR analyses were conducted (Figure S6). The yield of the products was determined by ^{19}F NMR spectra.[S2]

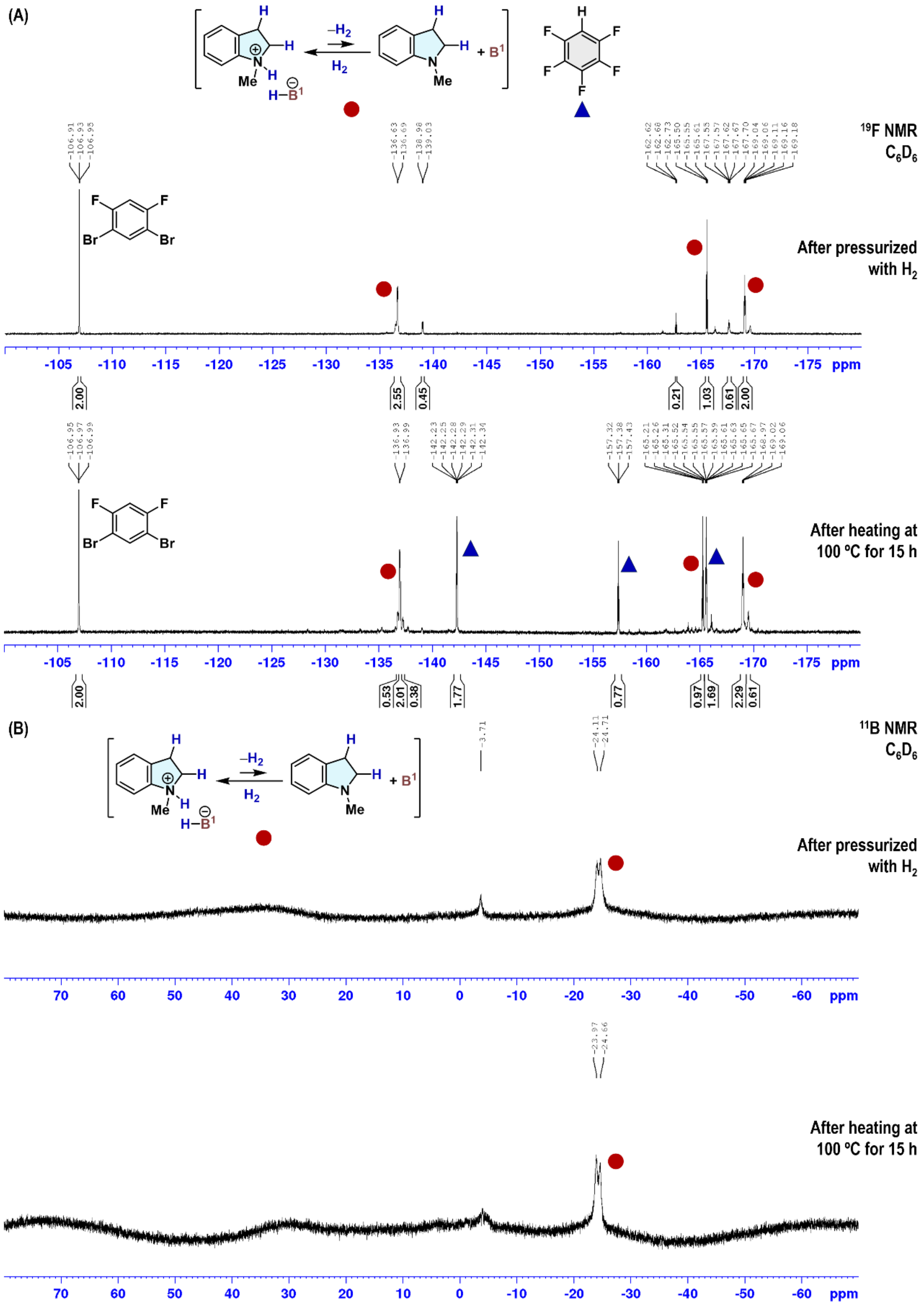
(A)
–H2
H2
+ B1
H–B1
19F NMR
C6D6
After pressurized with H2
After heating at 100 °C for 15 h
(B)
11B NMR
C6D6
After pressurized with H2
After heating at 100 °C for 15 h
ppm

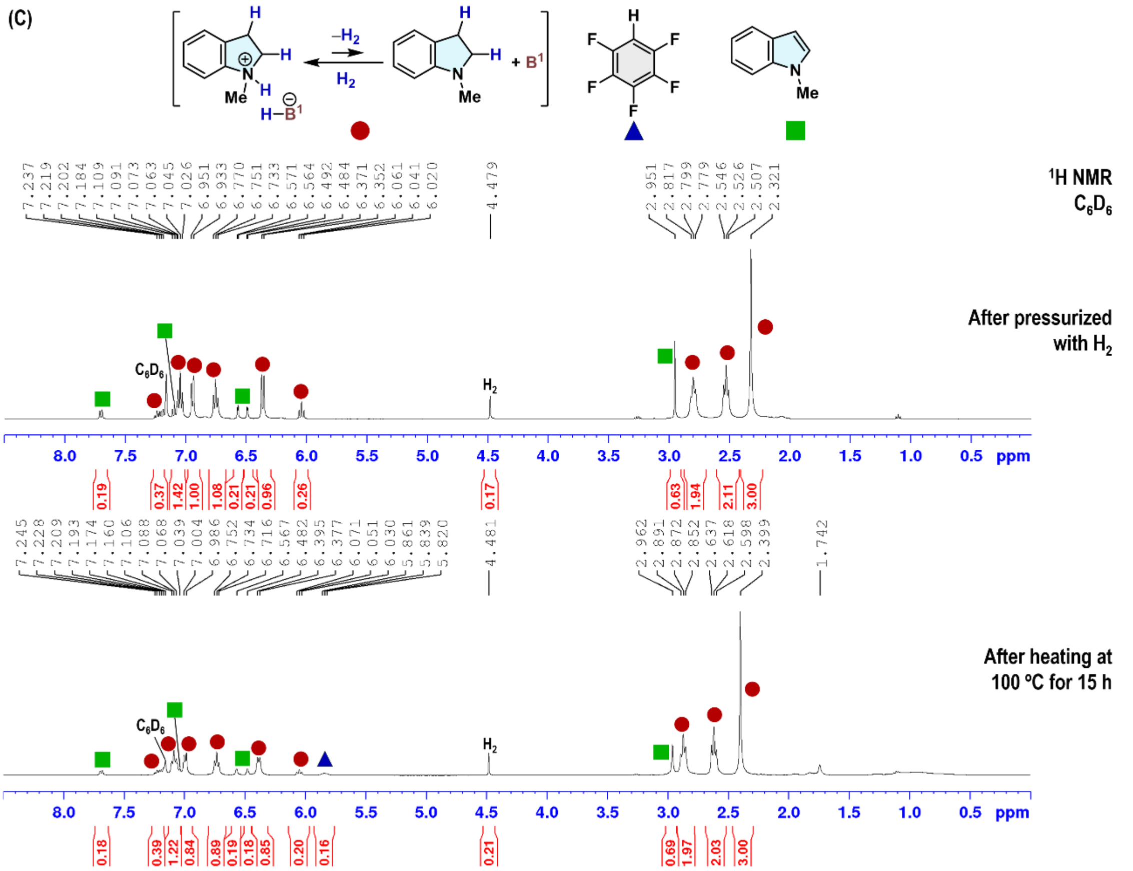

Figure S6. Stoichiometric reaction between $\mathbf{B^1}$ and **2a** in the presence of H_2. (A) ^{19}F NMR spectra. (B) ^{11}B NMR spectra. (C) ^1H NMR spectra.

[6] Scope of indoles

General: A 30 mL autoclave was charged with **1** (ca. 2.5 mmol) and $\mathbf{B}^n$ (n = 1 and/or 7; ca. 0.025 mmol; 1 mol%). Tetradecane or dodecane was added as an internal standard. Once sealed, the autoclave was pressurized with H_2 (20 atm) and heated at 100 °C for 16 h. After degassing at room temperature, the yield of **2** was determined by GC analysis.

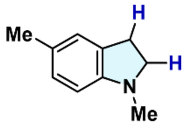

1,5-Dimethylindoline (2b): Followed by the general procedure, **1b** (369 mg; 2.54 mmol), $\mathbf{B^7}$ (17.8 mg; 0.0255 mmol), and tetradecane (244 mg; 1.23 mmol) were employed, giving **2b** in 93% GC yield. **^{1}H NMR** (400 MHz, $CDCl_3$): δ 6.94 (s, 1H, 4-C*H*), 6.91 (d, J = 8.0 Hz, 1H, 7-C*H*), 6.44 (d, J = 8.0 Hz, 1H, 6-C*H*), 3.26 (t, J = 8.0 Hz, 2H, 2-CH_2), 2.92 (t, J = 8.0 Hz, 2H, 3-CH_2), 2.74 (s, 3H, *N*-CH_3), 2.27 (s, 3H, 5-C-CH_3).[S3]

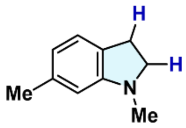

1,6-Dimethylindoline (2c): Followed by the general procedure, **1c** (360 mg; 2.48 mmol), $\mathbf{B^7}$ (17.5 mg; 0.0251 mmol), and tetradecane (236 mg; 1.19 mmol)

were employed, giving **2c** in 96% GC yield. ^{1}**H NMR** (400 MHz, $CDCl_3$): δ 6.89 (d, J = 7.2 Hz, 1H, 4-C*H*), 6.42 (d, J = 7.2 Hz, 1H, 5-C*H*), 6.26 (s, 1H, 7-C*H*), 3.20 (t, J = 8.0 Hz, 2H, 2-CH_2), 2.82 (t, J = 8.0 Hz, 2H, 3-CH_2), 2.67 (s, 3H, *N*-CH_3), 2.23 (s, 3H, 6-C-CH_3).[S4]

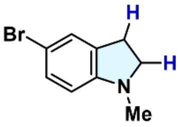

5-Bromo-1-methylindoline (2d): Followed by the general procedure, **1d** (574 mg; 2.73 mmol), **B**^{7} (17.5 mg; 0.0251 mmol), and tetradecane (238 mg; 1.20 mmol) were employed, giving **2d** in 96% GC yield. ^{1}**H NMR** (400 MHz, $CDCl_3$): δ 7.17–7.15 (m, 2H, 4-C*H*, 6-C*H*), 6.37 (d, J = 8.8 Hz 1H, 4-C*H*), 3.33 (t, J = 8.2 Hz, 2H, 2-CH_2), 2.93 (t, J = 8.2 Hz, 2H, 3-CH_2), 2.74 (s, 3H, *N*-CH_3).[S5]

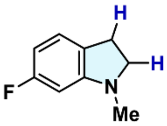

6-Fluoro-1-methylindoline (2e): Followed by the general procedure, **1e** (384 mg; 2.57 mmol), **B**^{7} (17.5 mg; 0.0251 mmol), and tetradecane (242 mg; 1.22 mmol) were employed, giving **2e** in 98% GC yield. ^{1}**H NMR** (400 MHz, $CDCl_3$): δ 6.96–6.93 (m, 1H, 4-C*H*), 6.34–6.29 (m, 1H, 5-C*H*), 6.16 (dd, $^{3}J_{H,F}$ = 10 Hz, $^{4}J_{H,H}$ = 2.0 Hz, 1H, 7-C*H*), 3.36 (t, J = 8.2 Hz, 2H, 2-CH_2), 2.90 (t, J = 8.2 Hz, 2H, 3-CH_2), 2.74 (s, 3H, *N*-CH_3).[S4]

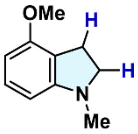

4-Methoxy-1-methylindoline (2f): Followed by the general procedure, **1f** (408 mg; 2.53 mmol), **B**^{7} (17.6 mg; 0.0252 mmol), and tetradecane (250 mg; 1.26 mmol) were employed, giving **2f** in 92% GC yield. ^{1}**H NMR** (400 MHz, $CDCl_3$): δ 7.08 (t, J = 8.0 Hz, 1H, 6-C*H*), 6.30 (d, J = 8.4 Hz, 1H, 7-C*H*), 6.20 (d, J = 7.6 Hz, 1H, 5-C*H*), 3.83 (s, 3H, *O*-CH_3), 3.32 (t, J = 8.4 Hz, 2H, 2-CH_2), 2.92 (t, J = 8.0 Hz, 2H, 3-CH_2), 2.76 (s, 3H, *N*-CH_3).[S6]

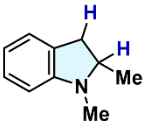

1,2-Dimethylindoline (2g): Followed by the general procedure, **1g** (364 mg; 2.51 mmol), **B**^{7} (17.6 mg; 0.0252 mmol), and dodecane (240 mg; 1.41 mmol) were employed, giving **2g** in 74% GC yield. ^{1}**H NMR** (400 MHz, $CDCl_3$): δ 7.16-7.09 (m, 2H, 4-C*H*, 6-C*H*), 6.72 (t, J = 7.2 Hz, 1H, 5-C*H*), 6.51 (d, J = 7.6 Hz, 1H, 7-C*H*), 3.48-3.42 (m, 1H, 2-CH_2), 3.16-3.10 (m, 1H, 3-CH_2), 2.77 (s, 3H, *N*-CH_3), 2.69-2.62 (m, 1H, 3-CH_2), 1.38 (d, J = 6.0 Hz, 3H, 2-C-CH_3).[S7]

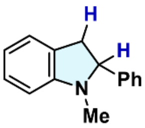

2-Phenyl-1-methylindoline (2h): Followed by the general procedure, **1h** (518 mg; 2.50 mmol), **B**^{1} (12.7 mg; 0.0248 mmol), and tetradecane (291 mg; 1.47 mmol) were employed, giving **2h** in 29% GC yield. ^{1}**H NMR** (400 MHz, $CDCl_3$): δ 7.37 (t, J = 8.0 Hz, 2H, 2-C-Ar-*H*), 7.26 (d, J = 8.0 Hz, 2H, 2-C-Ar-*H*), 7.16-7.21 (m, 2H, 4-C*H*, 7-C*H*), 7.10 (t, J = 7.6 Hz, 1H, 2-C-Ar-*H*), 6.99 (t, J = 7.2 Hz, 1H, 6-C*H*), 6.78 (t, J = 7.6 Hz, 1H, 5-C*H*), 3.98 (t, J = 8.4 Hz, 2H, 2-CH_2), 3.15 (t, J = 8.4 Hz, 2H, 3-CH_2).[S8]

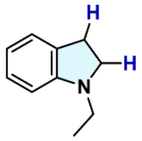

1-Ethylindoline (2i): Followed by the general procedure, **1i** (365 mg; 2.51 mmol), **B**^{7} (17.3 mg; 0.0248 mmol), and tetradecane (245 mg; 1.23 mmol) were employed, giving **2i** in 90% GC yield. **^{1}H NMR** (400 MHz, $CDCl_3$): δ 6.98 (t, J = 7.8 Hz, 2H, 5-CH, 6-CH), 6.56 (d, J = 7.2 Hz, 1H, 7-CH), 6.40 (d, J = 7.6 Hz, 1H, 4-CH), 3.24 (t, J = 8.0 Hz, 2H, 2-CH), 3.06 (q, J = 7.2 Hz, 2H, N-CH_2-CH_3), 2.87 (t, J = 8.0 Hz, 2H, 3-CH), 1.11 (t, J = 7.2 Hz, 3H, N-CH_2-CH_3).[S9]

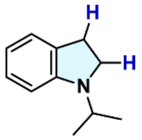

1-Isopropylindoline (2j): Followed by the general procedure, **1j** (413 mg; 2.59 mmol), **B**^{7} (17.6 mg; 0.0252 mmol), and tetradecane (242 mg; 1.22 mmol) were employed, giving **2j** in 78% GC yield. **^{1}H NMR** (400 MHz, $CDCl_3$): δ 7.05-7.08 (m, 2H, 4-CH, 6-CH), 6.62 (t, J = 7.2 Hz, 1H, 5-CH), 6.45 (d, J = 8.0 Hz, 1H, 7-CH), 3.80-3.90 (m, 1H, N-CH), 3.36 (t, J = 8.4 Hz, 2H, 2-CH_2), 2.96 (t, J = 8.4 Hz, 2H, 3-CH_2), 1.18 (d, J = 6.8 Hz, 6H, N-C-(CH_3)$_2$).[S10]

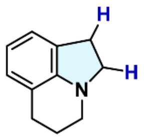

1,2,5,6-Tetrahydro-4H-pyrrolo[3,2,1-ij]quinoline (2k): Followed by the general procedure, **1k** (394 mg; 2.51 mmol), **B**^{7} (17.6 mg; 0.0252 mmol) and tetradecane (229 mg; 1.15 mmol) were employed, giving **2k** in 94% GC yield. **^{1}H NMR** (400 MHz, $CDCl_3$): δ 6.96 (d, J = 7.2 Hz, 1H, 4-CH), 6.85 (d, J = 7.2 Hz, 1H, 6-CH), 6.65 (t, J = 7.2 Hz, 1H, 5-CH), 3.28 (t, J = 7.8 Hz, 2H, 2-CH_2), 3.01 (t, J = 5.2 Hz, 2H, N-CH_2), 2.93 (t, J = 7.8 Hz, 2H, 3-CH_2), 2.72 (t, J = 6.6 Hz, 2H, 7-C-CH_2), 2.12 (m, 2H, N-CH_2-CH_2).[S11]

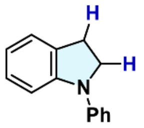

1-Phenylindoline (2l): Followed by the general procedure, **1l** (494 mg; 2.56 mmol), **B**^{1} (12.8 mg, 0.0250 mmol), **B**^{7} (17.7 mg; 0.0253 mmol) and tetradecane (250 mg; 1.26 mmol) were employed, giving **2l** in 94% GC yield. **^{1}H NMR** (400 MHz, $CDCl_3$): δ 7.46 (d, J = 7.2 Hz, 2H, 2-C-Ar-o-H_2), 7.38 (t, J = 7.4 Hz, 2H, 2-C-Ar-m-H_2), 7.32 (d, J = 7.2 Hz, 1H, 2-C-Ar-p-H), 7.15 (t, J = 7.6 Hz, 1H, 5-CH), 7.08 (d, J = 7.2 Hz, 1H, 4-CH), 6.73 (t, J = 7.5 Hz, 1H, 5-CH), 6.54 (d, J = 8.0 Hz, 1H, 7-CH), 4.36 (dd, J = 11.2, 8.8 Hz, 1H, 3-CH_2), 3.33 (dd, J = 15.6, 8.8 Hz, 1H, 3-CH_2), 2.94 (dd, J = 15.6, 11.2 Hz, 1H, 2-CH_2), 2.62 (s, 3H, N-CH_3).[S12]

We also explored the following Conditions 1-3 for the synthesis of **2l**;

Conditions 1: Followed by the general procedure, **1l** (506 mg; 2.62 mmol), **B**^{7} (35.0 mg; 0.0501 mmol; 2 mol%), **2a** (6.5 mg; 0.048 mmol; 2 mol%) and tetradecane (235 mg; 1.18 mmol) were employed, giving **2l** in 98% GC yield.

Conditions 2: Followed by the general procedure, **1l** (265 mg; 1.37 mmol), **B**^{7} (17.4 mg; 0.0249 mmol; 2 mol%) and tetradecane (242 mg; 1.22 mmol) were employed, giving **2l** in 15% GC yield.

Conditions 3: Followed by the general procedure, **1l** (242 mg; 1.25 mmol), **B**^{1} (13.2 mg; 0.0258

mmol; 2 mol%) and tetradecane (229 mg; 1.15 mmol) were employed, giving **2l** in 87% GC yield.

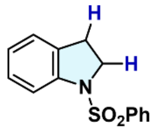

1-Phenylsulfonylindoline (2m): Followed by the general procedure, **1m** (644 mg; 2.50 mmol), **B**[1] (13.0 mg; 0.0254 mmol) and tetradecane (240 mg; 1.21 mmol) were employed, giving **2m** in 47% GC yield. **^{1}H NMR** (400 MHz, $CDCl_3$): δ 7.79 (d, J = 7.6 Hz, 2H, *S*-*o*-ArH_2), 7.65 (d, J = 8.0 Hz, 1H, *S*-*p*-Ar*H*), 7.55 (t, J = 7.2 Hz, 1H, 6-C*H*), 7.44 (t, J = 7.6 Hz, 2H, *S*-*m*-Ar*H*), 7.19 (d, J = 6.4 Hz, 1H, 4-C*H*), 7.08 (d, J = 7.2 Hz, 1H, 7-C*H*), 6.98 (t, J = 7.6 Hz, 1H, 5-CH_3), 3.93 (t, J = 8.4 Hz, 2H, 2-CH_2), 2.88 (t, J = 8.4 Hz, 2H, 3-CH_2).[S13]

[7] Theoretical studies

7-1. Computational details

The density functional theory (DFT) calculations were performed with Gaussian 16 (Revision C.01) software.[S14] All the structures were optimized at the M06-2X level of theory with the Def2-SVP basis set.[S15] Frequency calculations were performed to verify that intermediates have no imaginary frequency, whereas the transition state structures have only one imaginary frequency. The appropriateness of the connections between each reactant and product via the transition state was confirmed using intrinsic reaction coordinate (IRC).[S16] Single-point energy calculations were carried out at M06-2X/Def2-TZVP/gas-phase level of theory.[S17] The reported Gibbs free energies were calculated at 298.15 K. These calculations involve a certain margin of error.

7-2. Selected frontier molecular orbitals (FMOs) for [(*C3*-1a)···B^{1}]

Selected FMOs for **[(*C3*-1a)···B^{1}]** (from HOMO−2 to LUMO+3), calculated at M06-2X/Def2-TZVP//M06-2X/Def2-SVP level, are shown in Figure S7.

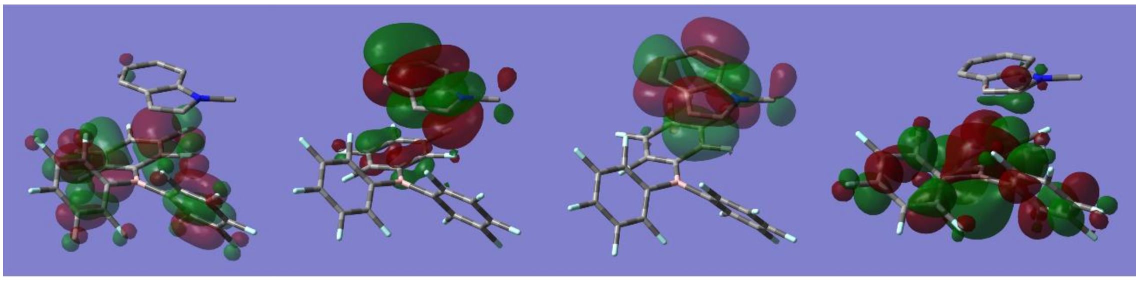

HOMO−2 (−8.82 eV) | HOMO−1 (−7.76 eV) | HOMO (−7.26 eV) | LUMO (−2.47 eV)

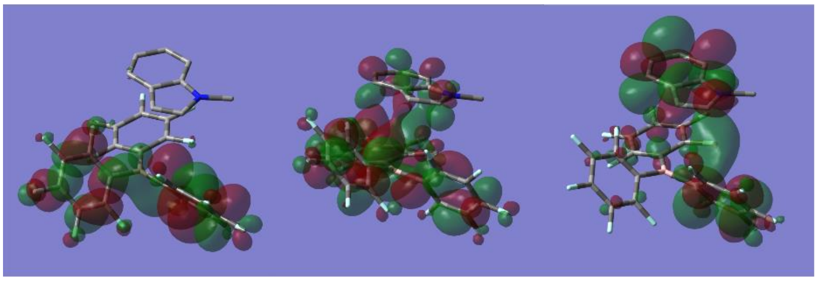

LUMO+1 (−0.39 eV) | LUMO+2 (−0.28 eV) | LUMO+3 (−0.03 eV)

Figure S7. Selected FMOs for **[(*C3*-1a)···B^{1}]**.

7-3. Plausible pathways for the reaction among 1a, B^1 and H_2

Plausible pathways for the reaction among **1a, B^1** and H_2 are shown in Figure 3C. The molecular structures of related compounds are summarized in Table S2.

Table S2. DFT-optimized structures in Figure 3C.

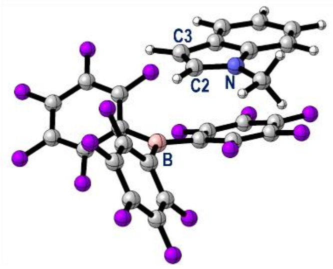 **[(*C3*-1a)···B^1]**	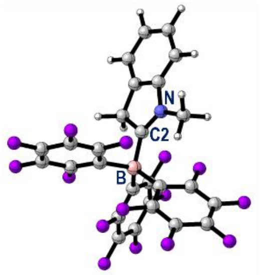 **(*C2*-1a)·B^1**	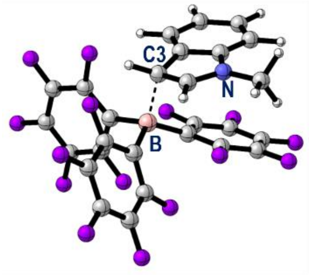 **(*C3*-1a)·B^1**
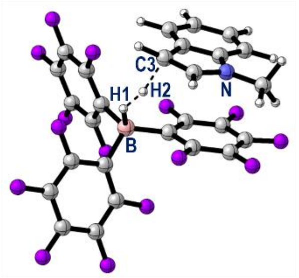 **TS1$_{(C3\text{-}B1)}$**	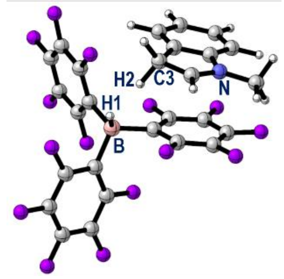 **[(*C3*-1a)–H][H–B^1]**	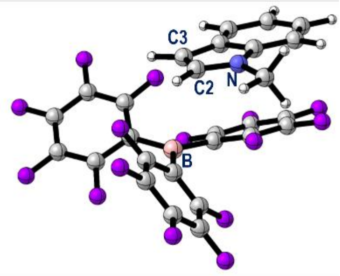 **[(*N*-1a)···B^1]**
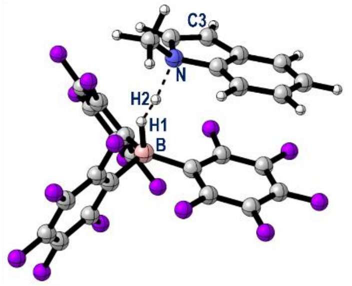 **TS1$_{(N\text{-}B1)}$**	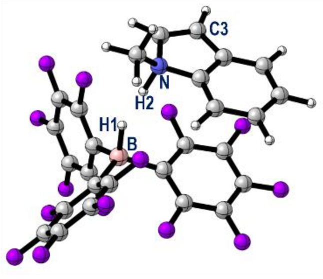 **[(*N*-1a)–H][H–B^1]**	

7-4. Plausible reaction mechanisms for the hydrogenation of 1a with B^7

Plausible reaction mechanisms for the hydrogenation of **1a** with $\mathbf{B^7}$ are shown in Figure 5. The molecular structures of related compounds are summarized in Table S3.

Table S3. DFT-optimized structures in Figure 5.

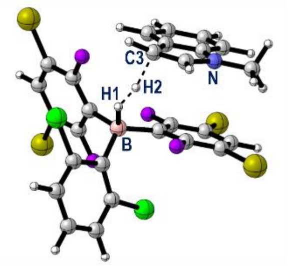<br>**TS1$_{(C3\text{-}B7)}$**	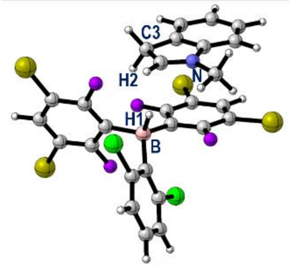<br>**[(*C3*-1a)–H][H–B^7]**	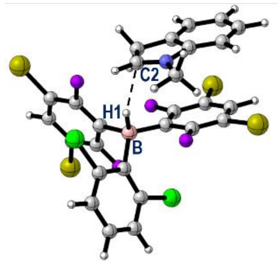<br>**TS2**
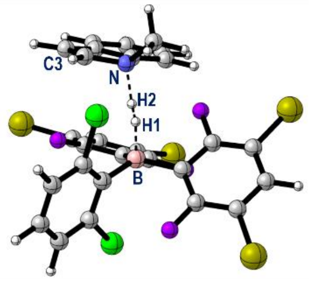<br>**TS1$_{(N\text{-}B7)}$**	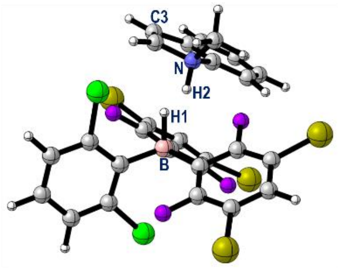<br>**[(*N*-1a)–H][H–B^7]**	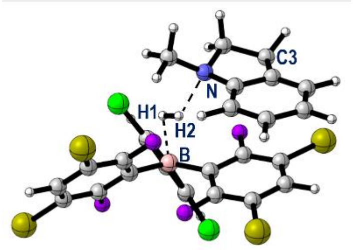<br>**TS3**
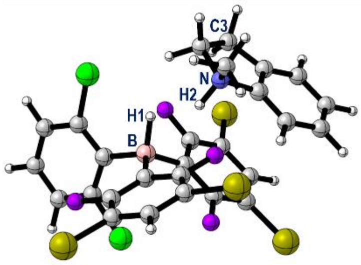<br>**[2a–H][H–B^7]**	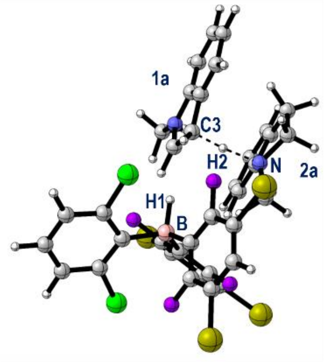<br>**TS4**	

[8] References for the Supporting Information

1. Dong, Y.; Lan, M. F.; Lin, Y. Q.; Chen, L.; Wu, C. M.; Wang, Z. F.; Shi, Z. C.; Deng, G. W.; He, B. *J. Org. Chem.* **2024**, *89*, 6474–6488.
2. Zhang, S.; Han, Y.; He, J.; Zhang, Y. *J. Org. Chem.* **2018**, *83*, 1377–1386.
3. Kato, S.; Saga, Y.; Kojima, M.; Fuse, H.; Matsunaga, S.; Fukatsu, A.; Kondo, M.; Masaoka, S.; Kanai, M. *J. Am. Chem. Soc.* **2017**, *139*, 2204–2207.
4. Han, Y.; Zhang, S.; He, J.; Zhang, Y. *J. Am. Chem. Soc.* **2017**, *139*, 7399–7407.
5. Xu, Z. M.; Hu, Z.; Huang, Y.; Bao, S. J.; Niu, Z.; Lang, J. P.; Al-Enizi, A. M.; Nafady, A.; Ma, S. *J. Am. Chem. Soc.* **2023**, *145*, 14994–15000.
6. Maiti, S.; Kim, J. S.; Kim, J. *New J. Chem.* **2024**, *48*, 3342–3346.
7. Li, W. D.; Zhu, D. Y.; Li, G.; Chen, J.; Xia, J. B. *Adv. Synth. Catal.* **2019**, *361*, 5098–5104.
8. Pandey, G.; Tiwari, S. K.; Singh, B. *Tetrahedron Lett.* **2016**, *57*, 4480–4483.
9. Gui, J.; Xie, H.; Jiang, H.; Zeng, W. *Org. Lett.* **2019**, *21*, 2804–2807.
10. Johnston, J. N.; Plotkin, M. A.; Viswanathan, R.; Prabhakaran, E. N. *Org. Lett.* **2001**, *3*, 1009–1011.
11. Mayer, R. J.; Hampel, N.; Mayer, P.; Ofial, A. R.; Mayr, H. *European J. Org. Chem.* **2019**, *2*, 412–421.
12. Xia, Q.; Tian, H.; Dong, J.; Qu, Y.; Li, L.; Song, H.; Liu, Y.; Wang, Q. *Chem. Eur. J.* **2018**, *24*, 9269–9273.
13. Poeira, D. L.; Macara, J.; Faustino, H.; Coelho, J. A. S.; Gois, P. M. P.; Marques, M. M. B. *European J. Org. Chem.* **2019**, *15*, 2695–2701.
14. Gaussian 16, Revision C.01, Frisch, M. J.; Trucks, G. W.; Schlegel, H. B.; Scuseria, G. E.; Robb, M. A.; Cheeseman, J. R.; Scalmani, G.; Barone, V.; Petersson, G. A.; Nakatsuji, H.; Li, X.; Caricato, M.; Marenich, A. V.; Bloino, J.; Janesko, B. G.; Gomperts, R.; Mennucci, B.; Hratchian, H. P.; Ortiz, J. V.; Izmaylov, A. F.; Sonnenberg, J. L.; Williams-Young, D.; Ding, F.; Lipparini, F.; Egidi, F.; Goings, J.; Peng, B.; Petrone, A.; Henderson, T.; Ranasinghe, D.: Zakrzewski, V. G.; Gao, J.; Rega, N.; Zheng, G.; Liang, W.; Hada, M.; Ehara, M.; Toyota, K.; Fukuda, R.; Hasegawa, J.; Ishida, M.; Nakajima, T.; Honda, Y.; Kitao, O.; Nakai, H.; Vreven, T.; Throssell, K.; Montgomery Jr., J. A.; Peralta, J. E.; Ogliaro, F.; Bearpark, M. J.; Heyd, J. J.; Brothers, E. N.; Kudin, K. N.; Staroverov, V. N.; Keith, T. A.; Kobayashi, R.; Normand, J.; Raghavachari, K.; Rendell, A. P.; Burant, J. C.; Iyengar, S. S.; Tomasi, J.; Cossi, M.; Millam, J. M.; Klene, M.; Adamo, C.; Cammi, R.; Ochterski, J. W.; Martin, R. L.; Morokuma, K.; Farkas, O.; Foresman, J. B.; Fox, D. J. Gaussian, Inc., Wallingford CT, 2019.
15. Schäfer, A.; Horn, H.; Ahlrichs, R. *J. Chem. Phys.* **1992**, *97*, 2571–2577.
16. Fukui, K. *Acc. Chem. Res.* **1981**, *14*, 363–368.
17. Schäfer, A.; Huber, C.; Ahlrichs, R. *J. Chem. Phys.* **1994**, *100*, 5829–5835.
